# Expression of Epithelial Cell Adhesion Molecule (EpCAM) in Oral Squamous Cell Carcinoma and Its Correlation With Clinicopathological Features: A Pilot Study From Northeast India

**DOI:** 10.7759/cureus.107321

**Published:** 2026-04-19

**Authors:** Pillar K Mylliemngap, Anupam Gogoi, Projnan Saikia, Hiranya P Saikia

**Affiliations:** 1 Pathology, Jorhat Medical College and Hospital, Jorhat, IND; 2 Otolaryngology, Assam Medical College and Hospital, Dibrugarh, IND

**Keywords:** biomarker, epithelial cell adhesion molecule, immunohistochemistry staining, northeast india, oral cavity squamous cell carcinoma

## Abstract

Background: Oral squamous cell carcinoma (OSCC) poses a major public health burden in South Asia, particularly in Northeast India, where exposure to smokeless tobacco and areca nut is highly prevalent. Epithelial cell adhesion molecule (EpCAM), a transmembrane glycoprotein involved in tumour proliferation and signalling pathways, has been implicated in the pathogenesis across multiple epithelial malignancies. This pilot study aimed to evaluate the immunohistochemical expression pattern of EpCAM in OSCC and to explore its association with clinicopathological parameters.

Methods: A hospital-based cross-sectional pilot study was conducted at the Department of Pathology, Jorhat Medical College and Hospital, Jorhat, Assam, India, from December 2024 to November 2025. Thirty-five biopsy-confirmed cases of OSCC were included. All specimens underwent routine haematoxylin and eosin (H&E) histopathological examination, followed by manual immunohistochemistry (IHC) staining with a monoclonal anti-EpCAM antibody (anti-EpCAM, clone BerEP4, PathnSitu, prediluted, ready to use). Staining was scored using a composite score (intensity 0-3 x proportion 0-4; total 0-12), with overexpression defined as a total score >4. Statistical associations were examined using Fisher's exact test (Statistical Product and Service Solutions (SPSS, version 26.0; IBM SPSS Statistics for Windows, Armonk, NY); significance level α = 0.05. Effect sizes were estimated using odds ratios (OR) with 95% confidence intervals (CI).

Results: The study population included 21 males and 14 females (male-to-female ratio 1.5:1), with a mean age of 54.8 years and a peak age group of 51-60 years. Buccal mucosa was the most frequent tumour site (37%), followed by alveolar mucosa (23%) and tongue (20%). Histologically, 24 cases (68.6%) were well-differentiated, 9 (25.7%) moderately differentiated, and 2 (5.7%) poorly differentiated. EpCAM overexpression (total score: >4) was observed in 10 of 35 cases (28.6%), weak expression (score: 1-4) in 12 cases (34.3%), and no expression in 13 cases (37.1%). Overall, EpCAM overexpression was observed in 28.6% of cases. A significant association was found between EpCAM overexpression and histological grade (p = 0.008), with moderately and poorly differentiated tumours demonstrating higher odds of overexpression compared to well-differentiated tumours (OR = 12.25; 95% CI: 2.18-68.6). Among other clinicopathological parameters, overexpression was more frequently observed in males (38.1% vs 14.3% in females), alcohol users (57.1% of exclusive alcohol users), and tongue carcinomas (57.1% of tongue cases); however, these associations were not statistically significant.

Conclusion: EpCAM overexpression was detected in approximately 28.6% of OSCC cases in this Northeast Indian cohort. The observed trends suggest increased EpCAM overexpression in males, those with alcohol consumption, and tongue carcinomas, while a significant association was observed with higher histological grade. This pilot study is limited by a small sample size and a lack of TNM staging and survival data, restricting prognostic interpretation. These findings should be considered preliminary and exploratory in nature. Larger prospective multi-centre studies are needed to validate these observations and determine the prognostic and therapeutic relevance of EpCAM in OSCC.

## Introduction

Squamous cell carcinoma (SCC) of the head and neck region ranks as the sixth most common malignancy globally, with oral SCC (OSCC) comprising a substantial proportion of these cases [1]. Globally, approximately 377,000 new cases and 177,000 deaths from lip and oral cavity cancers are recorded annually [1]. India carries a disproportionately high burden of head and neck cancers, contributing substantially to the global incidence, with national registry data reporting over 75,000 new cases annually [2-4]. The Northeast Indian states have particularly high incidence rates attributable to culturally entrenched practices of areca nut, betel quid, and smokeless tobacco chewing from an early age [4,5].

Despite medical advances, the five-year survival rate for OSCC has remained stagnant at approximately 50-60% over the past four decades, with poor outcomes particularly in locally advanced and poorly differentiated disease [1,6]. This underscores the need for reliable biomarkers capable of identifying high-risk tumours and developing targeted therapeutic interventions.

Epithelial cell adhesion molecule (EpCAM) is a type I transmembrane glycoprotein of 39-42 kDa encoded by the *EPCAM *gene on chromosome 2p21. EpCAM comprises an extracellular domain (EpEX), a single transmembrane domain, and a short intracellular domain (EpICD). Under physiological conditions, EpCAM mediates homotypic epithelial cell adhesion [7]. In neoplastic states, regulated intramembrane proteolysis by ADAM10/ADAM17 and ϓ-secretase liberates EpICD, which then forms a nuclear transcriptional complex with *four-and-a-half LIM domain protein 2 *(*FHL2*), β-catenin, and lymphoid enhancer factor-1 (LEF-1), activating genes involved in cell proliferation (c-myc, cyclin D1), stemness (Nanog, Oct4), and invasion [7]. EpCAM overexpression has been reported in carcinomas of the breast, colon, lung, thyroid, and head and neck and has been associated with tumour progression, lymph node metastasis, and adverse prognosis [7,8-10].

In OSCC, EpCAM expression data remain limited and derived predominantly from small single-institution studies. Published studies have reported variable EpCAM overexpression rates in OSCC, generally ranging from approximately 18%-30% across different cohorts with heterogeneous associations with clinicopathological parameters [8-10]. The biological basis for site-specific and grade-specific EpCAM upregulation and its potential as a target for anti-EpCAM therapies (e.g., catumaxomab, adecatumumab) warrants further study, particularly in populations with distinct risk factor profiles, such as Northeast India [7].

Recent molecular research has increasingly highlighted EpCAM as an important regulator of cancer stem cell (CSC) biology in OSCC. EpCAM signalling has been implicated in tumour proliferation, therapeutic resistance, and maintenance of CSC populations, suggesting that its overexpression may contribute to tumour aggressiveness and disease progression. These findings have renewed interest in EpCAM as a potential biomarker and therapeutic target in OSCC [9,10].

What remains unclear is whether EpCAM expression patterns are influenced by region-specific carcinogenic exposures and whether its clinicopathological associations are reproducible in high-risk populations such as Northeast India.

This pilot cross-sectional study aimed primarily to evaluate the immunohistochemical expression pattern of EpCAM in OSCC biopsies from Northeast India. Secondary objectives were to explore its association with clinicopathological parameters, including age, sex, carcinogen exposure, anatomical tumour site, and histological grade. Given the pilot nature of the study, these analyses were exploratory and intended to generate hypotheses for future investigation.

We hypothesize that EpCAM expression may show distinct clinicopathological associations in this population, potentially reflecting region-specific carcinogenic exposures.

## Materials and methods

Study design and setting

This hospital-based, cross-sectional study was conducted as an exploratory pilot investigation at the Department of Pathology, Jorhat Medical College and Hospital (JMCH), Jorhat, Assam, India, from December 2024 to November 2025, with the aim of generating preliminary data on EpCAM expression patterns in a high-risk population, assessing the feasibility of immunohistochemistry (IHC)-based scoring, and informing sample size estimation for future studies. A non-probability consecutive sampling technique was employed, whereby all histopathologically confirmed cases of OSCC presenting to JMCH for treatment during the study period were enrolled, provided that they fulfilled the predefined eligibility criteria. No a priori sample size or power calculation was performed as this was an exploratory pilot study.

The study population comprised 35 biopsy-confirmed cases of OSCC that visited JMCH for their treatment. Patients of all age groups and both sexes were eligible for inclusion, and written informed consent was obtained from each participant prior to enrolment. Cases were excluded if they involved non-squamous cell malignancies or pre-malignant lesions, benign oral conditions, recurrent disease or post-treatment specimens, or if the biopsy material was inadequate or non-representative for histopathological assessment. Ethical clearance was obtained from the Institutional Human Ethics Committee prior to commencement of the study (reference number: SMEJ/JMCH/MEU/841/Pt III/2023/4831, dated 18th November 2024).

Clinical data collection

Patient clinical data were collected, including age, sex, anatomical site of the primary tumour, and history of carcinogenic exposures (smokeless tobacco use, smoking, alcohol consumption, or combinations thereof). Exposure information was obtained from clinical records and/or patient history and recorded as categorical variables (present/absent). Quantitative details, such as duration, frequency, and cumulative exposure (e.g., pack-years or chewing frequency), were not available and were not included in the analysis. Tumour-node-metastasis (TNM) staging could not be assessed, as all specimens were derived from incisional or punch biopsies; this represents a recognized limitation of the present pilot study.

Histopathological examination

Specimens were fixed in 10% neutral buffered formalin, followed by dehydration in ascending grades of alcohol, clearing in xylene, paraffin embedding, and sectioning at 3-5 um thickness. Sections were stained with haematoxylin and eosin (H&E) and examined under light microscopy. Tumours were graded as well-differentiated SCC (WDSCC), moderately differentiated SCC (MDSCC), or poorly-differentiated SCC (PDSCC) based on conventional histopathological criteria as described in the literature [1].

Immunohistochemistry (IHC)

IHC was performed manually, using a polymer-based detection system, on 3-5 um sections cut from representative formalin-fixed paraffin-embedded (FFPE) blocks. Sections were dewaxed in xylene (two changes, 10 minutes each), rehydrated through graded alcohols to water, and subjected to antigen retrieval in citrate buffer (pH 6.0) using a pressure cooker. After blocking endogenous peroxidase with 3% hydrogen peroxide for 10 minutes, the sections were then incubated with the primary antibody (anti-EpCAM, clone BerEP4, PathnSitu, prediluted, ready to use) for 60 minutes at room temperature in a humidified chamber, followed by polymer-HRP secondary detection and visualisation using 3,3’-diaminobenzidine (DAB) chromogen. Sections were counterstained using Mayer's hematoxylin. Positive controls comprised sections of normal colonic mucosa and thyroid follicular epithelium, both of which demonstrate constitutive EpCAM membranous expression. Negative controls were processed identically with the omission of the primary antibody.

IHC scoring

All slides were evaluated using a whole-slide assessment approach, as the study was based on incisional and punch biopsy specimens with limited tumour area. The entire tumour present on the section was assessed rather than selecting hotspot fields. Both membranous and cytoplasmic staining were considered during evaluation, with greater emphasis on membranous localisation given the established role of EpCAM as a transmembrane adhesion molecule [7], though cytoplasmic staining has also been described in tumour tissues and may reflect intracellular processing and signalling activity [7-9]. Stained slides were evaluated independently by two pathologists blinded to clinicopathological data. Interobserver agreement for EpCAM was assessed using Cohen’s kappa coefficient; an initial concordance rate of 97.1% was achieved, yielding a Cohen's kappa (κ) of 0.932 (95% confidence interval (CI): 0.801-1.000). Staining was assessed for both the proportion of immunoreactive tumour cells (proportion score: 0 = none; 1 = 1-10%; 2 = 11-50%; 3 = 51-80%; 4 = >80%) and the intensity of staining (intensity score: 0 = absent; 1 = weak; 2= moderate; 3 = strong). A composite total score (range: 0-12) was calculated as the product of proportion and intensity scores. EpCAM overexpression was defined as a total score >4, weak expression as 1-4, and no expression as 0, as described by Spizzo et al. [11]. In cases of heterogeneous staining, scoring was based on the overall distribution of staining across the tumour, with proportion scores reflecting the estimated percentage of immunoreactive tumour cells. For statistical analysis, EpCAM expression status was dichotomised as overexpression (score: >4) versus non-overexpression (score: 0-4); descriptive data for the three expression categories (absent, weak, overexpression) are presented separately in the tables. The cutoff for EpCAM overexpression (total score: >4) was adopted from previously published studies [11] and has been applied in epithelial malignancies, including head and neck cancers. Although not specifically standardised for the present antibody clone, the use of this scoring framework allows comparability with the existing literature.

Statistical analysis

Statistical analysis was performed using Statistical Product and Service Solutions (SPSS, version 26.0; IBM SPSS Statistics for Windows, Armonk, NY) after data entry in Microsoft Excel (Microsoft® Corp., Redmond, WA). Categorical variables were expressed as frequencies and percentages. The association between EpCAM overexpression and each clinicopathological variable (sex, age group, habit, anatomical site, and histological grade) was assessed using Fisher's exact test, which was preferred over chi-square due to small sample size and low expected cell frequencies. Statistical significance was defined as a two-tailed p-value <0.05. Effect sizes were expressed as odds ratios (OR) with 95% CI for key comparisons. Given the small sample size and sparse data in several categories, analyses were considered exploratory in nature. Where appropriate, categories were combined to facilitate meaningful comparison. Formal regression modelling was not performed due to the limited sample size and the risk of unstable estimates.

## Results

Clinicopathological profile

**Figure 1:**
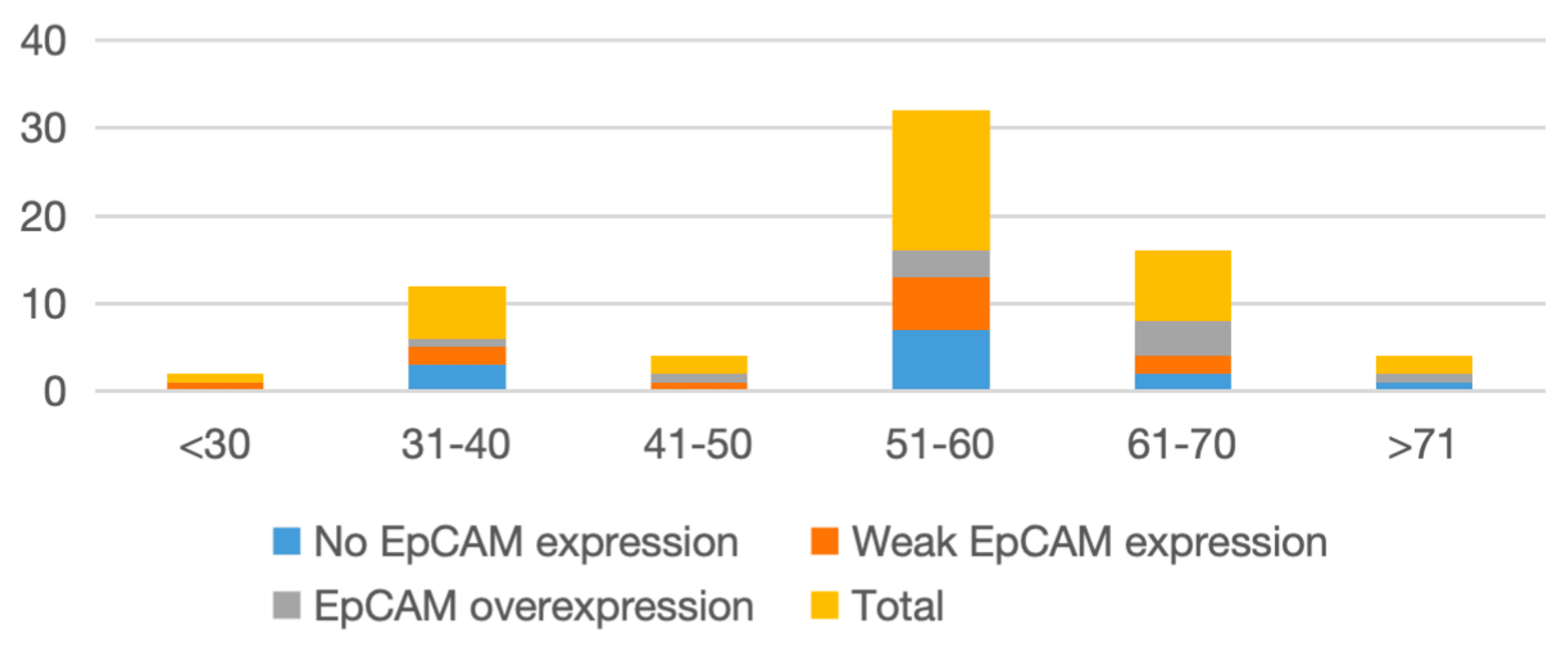
Age distribution in OSCC cases OSCC: oral squamous cell carcinoma; EpCAM: epithelial cell adhesion molecule

A total of 35 biopsy-confirmed cases of OSCC were evaluated in this study. The cohort showed a male predominance, with a male-to-female ratio of 1.5:1 and a mean age of 54.8 years. The majority of patients were clustered in the fifth and sixth decades of life. EpCAM overexpression did not demonstrate a uniform age-related trend. While the highest absolute number of cases was observed in the 51-60-year age group, overexpression was relatively more frequent in older age groups (≥60 years), where nearly half of the cases demonstrated increased expression. In contrast, younger age groups showed predominantly absent or weak expression. The age-wise distribution of cases and corresponding EpCAM expression patterns is shown in Table 1 and Figure 1.

**Table 1:** Age distribution in OSCC cases OSCC: oral squamous cell carcinoma; EpCAM: epithelial cell adhesion molecule

Age group (in years)	No EpCAM expression	Weak EpCAM expression	EpCAM overexpression	Total
<30	0	1	0	1
31-40	3	2	1	6
41-50	0	1	1	2
51-60	7	6	3	16
61-70	2	2	4	8
>70	1	0	1	2

**Figure 2:**
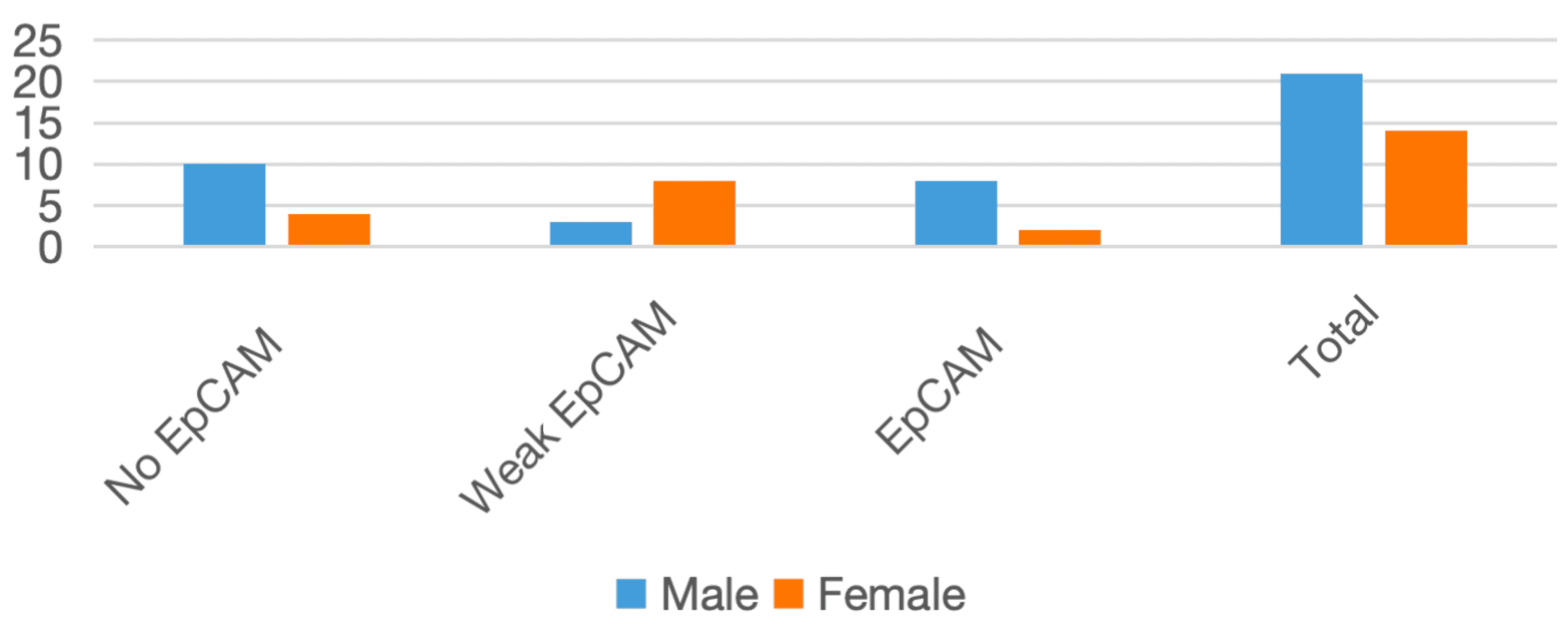
Gender-wise distribution in OSCC cases OSCC: oral squamous cell carcinoma; EpCAM: epithelial cell adhesion molecule

A male predominance was observed, with a higher proportion of EpCAM overexpression in males, whereas females largely exhibited weak or no expression. The gender distribution and EpCAM expression patterns are presented in Table 2 and Figure 2.

**Table 2:** Gender-wise distribution in OSCC cases OSCC: oral squamous cell carcinoma; EpCAM: epithelial cell adhesion molecule

Gender	No EpCAM expression	Weak EpCAM expression	EpCAM overexpression	Total
Male	9	4	8	21
Female	4	8	2	14

**Figure 3:**
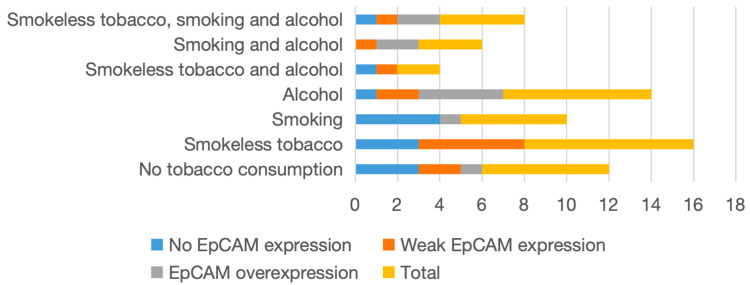
Distribution of OSCC cases based on habits OSCC: oral squamous cell carcinoma; EpCAM: epithelial cell adhesion molecule

A distinct variation in EpCAM expression was observed across different habit groups. Alcohol consumption, either alone or in combination with smoking, was associated with the highest proportion of EpCAM overexpression. In contrast, smokeless tobacco users predominantly demonstrated weak or absent expression, with no cases showing overexpression. This suggests a differential impact of carcinogenic exposures on EpCAM expression patterns. The distribution of cases based on carcinogenic habits is shown in Table 3 and Figure 3.

**Table 3:** Distribution of OSCC cases based on habits OSCC: oral squamous cell carcinoma; EpCAM: epithelial cell adhesion molecule

Habits	No EpCAM expression	Weak EpCAM expression	EpCAM overexpression	Total
No tobacco consumption	3	2	1	6
Smokeless tobacco	3	5	0	8
Smoking	4	0	1	5
Alcohol	1	2	4	7
Smokeless tobacco and alcohol	1	1	0	2
Smoking and alcohol	0	1	2	3
Smokeless tobacco, smoking and alcohol	1	1	2	4

**Figure 4:**
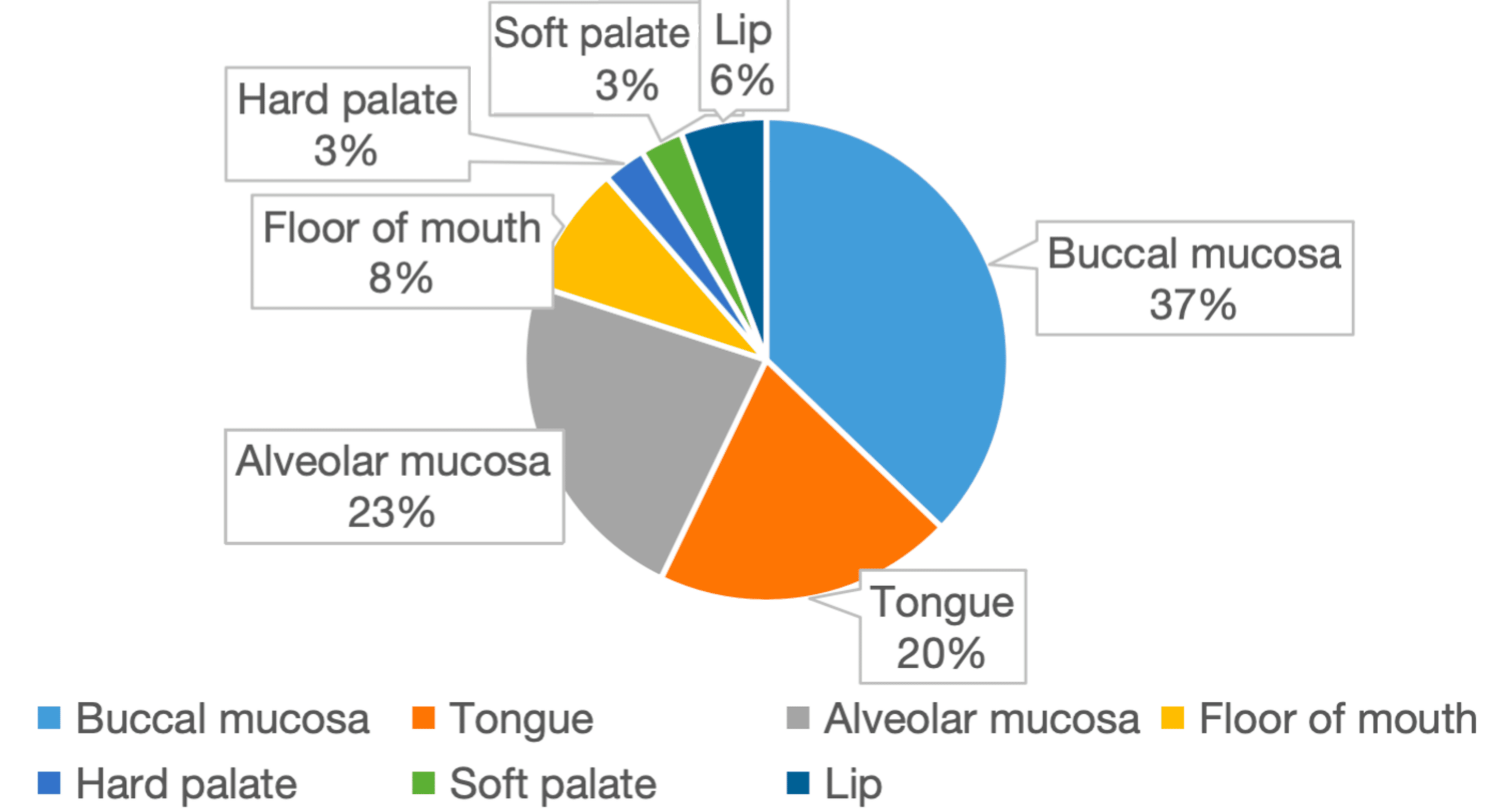
Distribution of OSCC cases based on anatomical sites OSCC: oral squamous cell carcinoma

Buccal mucosa was the most commonly involved site; however, EpCAM overexpression was disproportionately higher in tongue carcinomas. More than half of tongue lesions demonstrated overexpression, compared to relatively lower frequencies in buccal and alveolar mucosa. Other sites, such as the hard palate, showed no overexpression, whereas the single soft palate case demonstrated overexpression. This indicates a possible site-specific variation in EpCAM expression, although findings in less frequent sites should be interpreted with caution due to the small sample size. The distribution of tumour sites and corresponding EpCAM expression is summarised in Table 4 and Figure 4.

**Table 4:** Distribution of OSCC cases based on anatomical sites OSCC: oral squamous cell carcinoma; EpCAM: epithelial cell adhesion molecule

Site	No EpCAM expression	Weak EpCAM expression	EpCAM overexpression	Total
Buccal mucosa	4	7	2	13
Tongue	2	1	4	7
Alveolar mucosa	3	4	1	8
Floor of mouth	2	0	1	3
Hard palate	1	0	0	1
Soft palate	0	0	1	1
Lip	1	0	1	2

A clear gradation in EpCAM expression was observed with tumour differentiation, with increasing expression from well- to poorly differentiated tumours. Well-differentiated tumours predominantly exhibited absent or weak expression, whereas moderately differentiated tumours showed a higher proportion of overexpression. Notably, all poorly differentiated tumours demonstrated EpCAM overexpression. This pattern suggests an association between EpCAM expression and tumour dedifferentiation.

**Figure 5:**
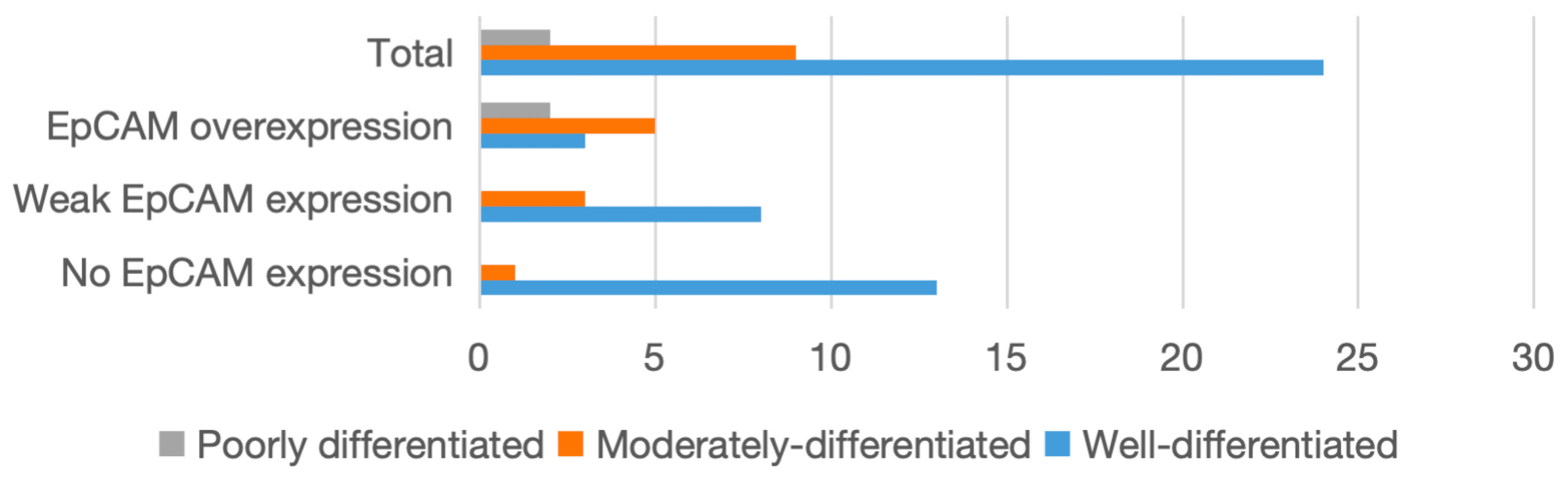
Distribution of OSCC cases based on morphological grades OSCC: oral squamous cell carcinoma; EpCAM: epithelial cell adhesion molecule

For effect size estimation, histological grades were grouped as well-differentiated and moderately/poorly differentiated tumours. The latter group demonstrated significantly higher odds of EpCAM overexpression versus well-differentiated tumours (OR = 12.25; 95% CI: 2.18-68.6), further supporting the trend of increasing EpCAM expression with higher tumour grade. The relationship between histological grade and EpCAM expression is shown in Table 5 and Figure 5.

**Table 5:** Distribution of OSCC cases based on morphological grades WDSCC: well-differentiated squamous cell carcinoma; MDSCC: moderately differentiated squamous cell carcinoma; PDSCC: poorly differentiated squamous cell carcinoma

Grade	No EpCAM expression	Weak EpCAM expression	EpCAM overexpression	Total
WDSCC	13	8	3	24
MDSCC	0	4	5	9
PDSCC	0	0	2	2

The majority of cases showed either absent or weak expression, with overexpression observed in approximately one-third of cases. High composite scores were primarily seen in tumours with higher grades and specific anatomical locations, indicating heterogeneity in expression across the cohort. The overall distribution of EpCAM expression categories is summarised in Table 6.

**Table 6:** Distribution of OSCC cases according to EpCAM staining OSCC: oral squamous cell carcinoma; EpCAM: epithelial cell adhesion molecule

EpCAM	Number	Percentage
No Expression (0)	13	37.1%
Weak Expression (1-4)	12	34.3%
Overexpression (>4)	10	28.6%
Total	35	100%

The association between EpCAM overexpression and clinicopathological parameters is summarised in Table 7. Overexpression was more frequent in males (8/21, 38%) than in females (2/14, 14%), though this difference did not reach statistical significance (p = 0.14, Fisher's exact test). No statistically significant association was found with age group (p = 0.52). Regarding habits, EpCAM overexpression was most frequent in exclusive alcohol users (4/7, 57%) and in those with combined smoking and alcohol use (2/3, 66.7%), while smokeless tobacco users showed no overexpression (0/8, 0%); the overall association was not statistically significant (p = 0.07).

**Table 7:** Association between EpCAM overexpression and clinicopathological parameters Fisher's exact test (two-tailed) was used for statistical analysis. A p-value of <0.05 was considered statistically significant. † = statistically significant values; n = number of cases; WDSCC = well-differentiated squamous cell carcinoma; MDSCC = moderately differentiated squamous cell carcinoma; PDSCC = poorly differentiated squamous cell carcinoma

Parameter	Total n	EpCAM overexpression n (%)	p-value
Sex: Male	21	8 (38%)	0.14
Sex: Female	14	2 (14%)	-
Age group (years)	35	-	0.52
<30	1	0 (0%)	-
31-40	6	1 (16.7%)	-
41-50	2	1 (50.0%)	-
51-60	16	3 (18.8%)	-
61-70	8	4 (50.0%)	-
>70	2	1 (50.0%)	-
Habits	35	-	0.07
No tobacco	6	1 (16.7%)	-
Smokeless tobacco	8	0 (0%)	-
Smoking only	5	1 (20.0%)	-
Alcohol only	7	4 (57.1%)	-
Smokeless + alcohol	2	0 (0%)	-
Smoking + alcohol	3	2 (66.7%)	-
All three	4	2 (50.0%)	-
Anatomical site	35	-	0.18
Buccal mucosa	13	2 (15.4%)	-
Tongue	7	4 (57.1%)	-
Alveolar mucosa	8	1 (12.5%)	-
Floor of mouth	3	1 (33.3%)	-
Hard palate	1	0 (0%)	-
Soft palate	1	1 (100%)	-
Lip	2	1 (50.0%)	-
Histological grade	35	-	0.008†
WDSCC	24	3 (12.5%)	-
MDSCC	9	5 (55.6%)	-
PDSCC	2	2 (100.0%)	-

Among anatomical sites, tongue carcinomas had the highest rate of overexpression (4/7, 57%), while buccal mucosa was the most frequent tumour site. The most striking association was observed with histological grade: overexpression rates were 12.5% in WDSCC, 55.6% in MDSCC, and 100% in PDSCC (p = 0.008, Fisher's exact test), representing a statistically significant finding in this study, though further studies are needed to validate this finding due to the small sample size of the present study.

The statistically significant association of EpCAM overexpression with histological grade (p = 0.008, Fisher's exact test) provides a biological context for the directional trends observed across the preceding parameters. The higher overexpression rates seen in alcohol users and tongue carcinomas are consistent with these subgroups harbouring a proportionately greater representation of higher-grade tumours. In contrast, the associations with sex (p = 0.14), carcinogen habit (p = 0.07), and anatomical site (p = 0.18) did not attain statistical significance, most likely reflecting the limited statistical power owing to the small sample size.

Photomicrographs 

**Figure 6:**
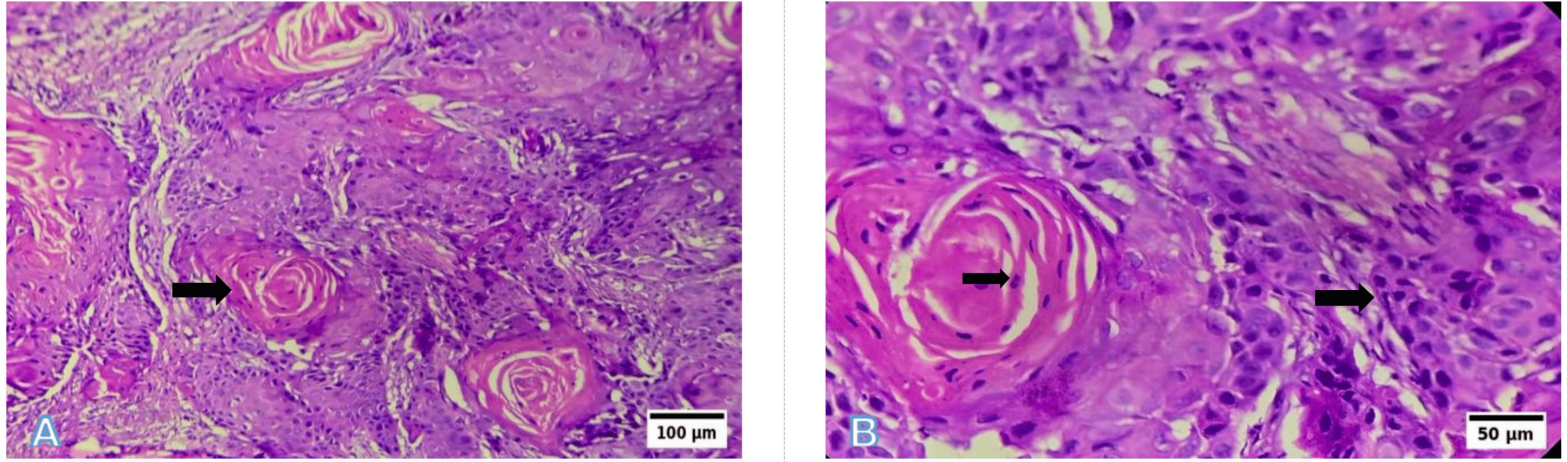
Representative photomicrographs of the histological grading for WDSCC (A) Low power (10×, H&E): Islands of well-differentiated squamous epithelium with prominent keratin pearl formation. (B) High power (40×, H&E): Individual cell keratinisation, intercellular bridges, and pleomorphic nuclei. Scale bars correspond to 100 µm in panel (A) and 50 µm in panel (B). WDSCC: well-differentiated squamous cell carcinoma; H&E: hematoxylin and eosin

**Figure 7:**
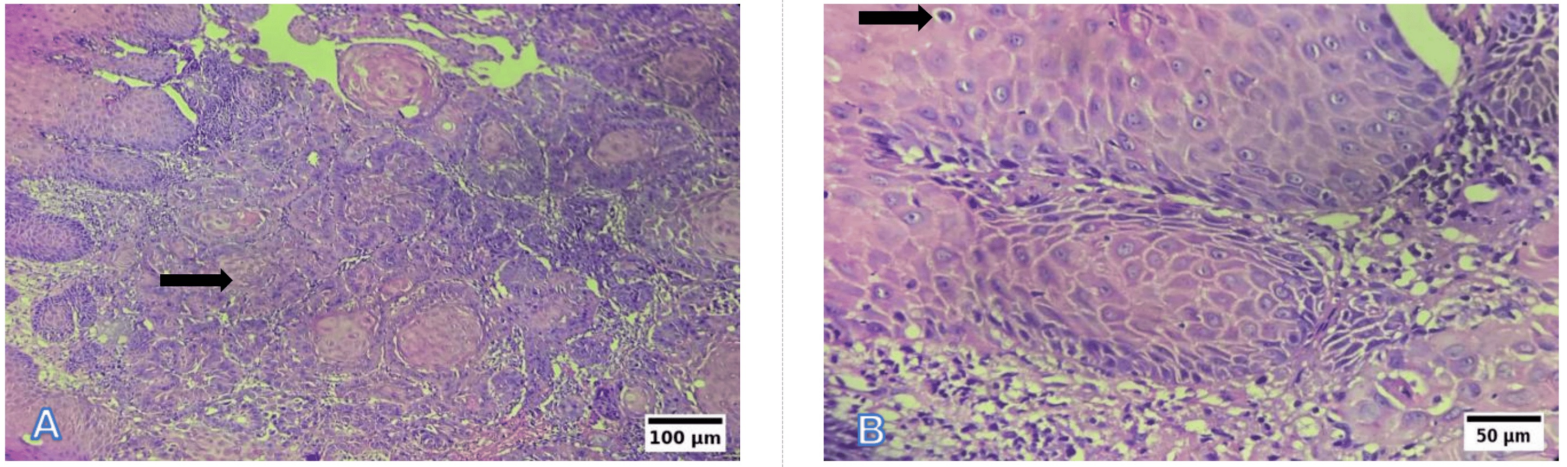
Representative photomicrographs of the histological grading for MDSCC (A) Low power (10×, H&E): Irregular tumour nests with reduced keratinisation. (B) High power (40×, H&E): Moderate nuclear pleomorphism and scattered mitoses. Scale bars correspond to 100 µm in panel (A) and 50 µm in panel (B). MDSCC: moderately differentiated squamous cell carcinoma; H&E: hematoxylin and eosin

**Figure 8:**
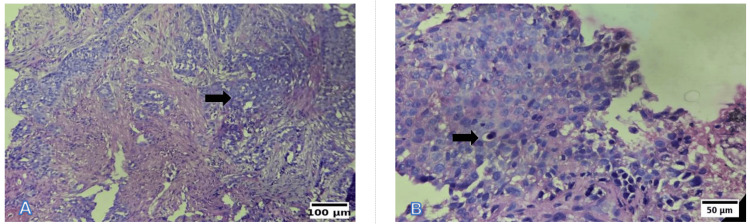
Representative photomicrographs of the histological grading for PDSCC (A) Low power (10×, H&E): Diffuse sheet-like infiltration with minimal keratinisation. (B) High power (40×, H&E): Markedly pleomorphic nuclei with brisk mitotic activity. Scale bars correspond to 100 µm in panel (A) and 50 µm in panel (B). PDSCC: poorly differentiated squamous cell carcinoma; H&E: hematoxylin and eosin

Representative photomicrographs of histological grading are shown in Figures 6-8. WDSCC is illustrated in Figure 6, demonstrating prominent keratin pearl formation and individual cell keratinisation. MDSCC, depicted in Figure 7, shows irregular tumour nests with reduced keratinisation and increased nuclear pleomorphism. PDSCC, as shown in Figure 8, is characterised by diffuse sheets of tumour cells with minimal keratinisation and marked pleomorphism.

**Figure 9:**
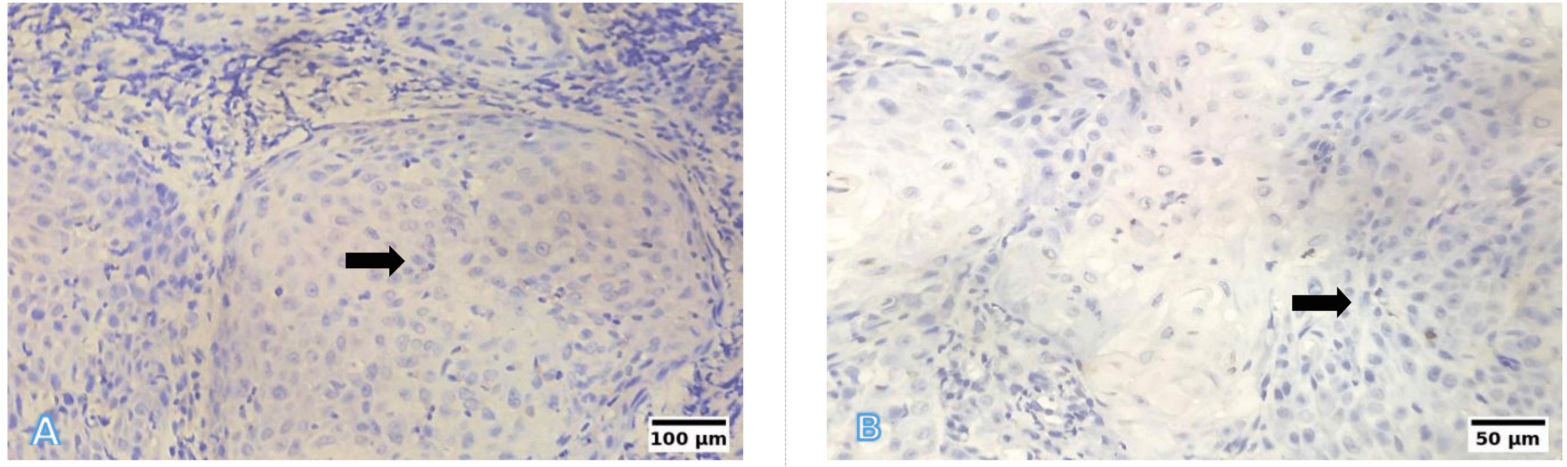
EpCAM immunohistochemistry: No expression (total score of 0) (A) 10×: Absent EpCAM expression in tumour cells (WDSCC). (B) 40×: Only background haematoxylin counterstain visible. Scale bars correspond to 100 µm in panel (A) and 50 µm in panel (B). EpCAM: epithelial cell adhesion molecule; WDSCC: well-differentiated squamous cell carcinoma

**Figure 10:**
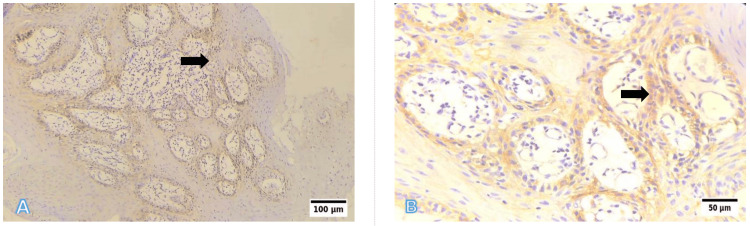
EpCAM immunohistochemistry: Weak expression (total score of 2; intensity 1 × proportion 2) (A) 10×: Focal weak membranous and cytoplasmic staining in WDSCC. (B) 40×: Weak membranous and cytoplasmic staining in 11-50% of tumour cells. Scale bars correspond to 100 µm in panel (A) and 50 µm in panel (B). EpCAM: epithelial cell adhesion molecule; WDSCC: well-differentiated squamous cell carcinoma

**Figure 11:**
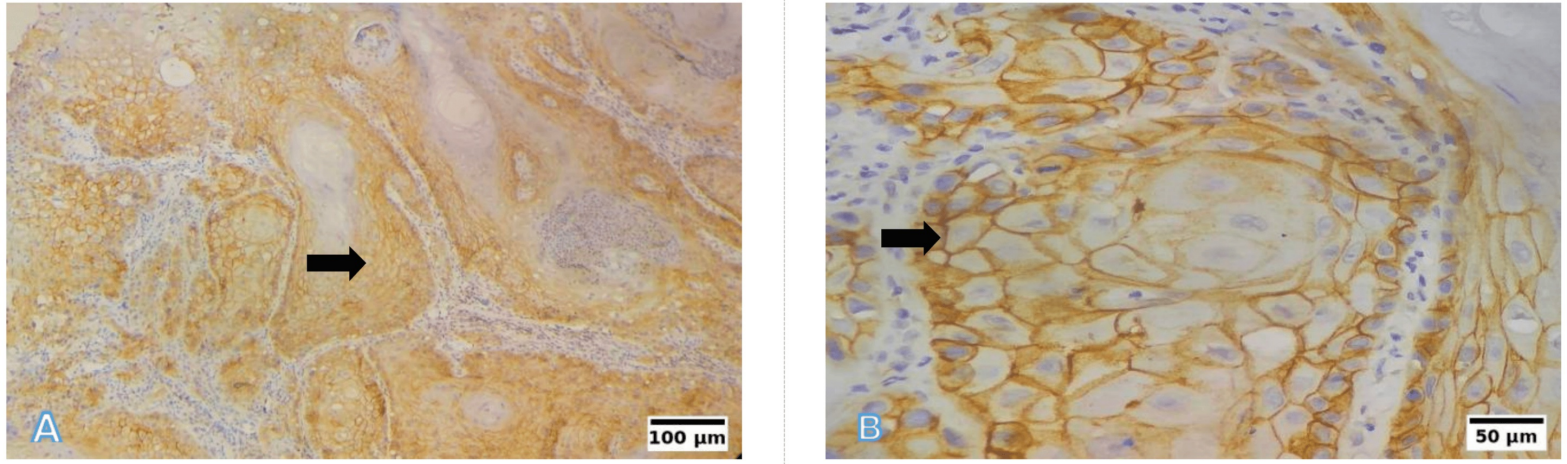
EpCAM immunohistochemistry: Overexpression (total score of 6; intensity 2 × proportion 3) (A) 10×: Diffuse moderate membranous staining in WDSCC. (B) 40×: Distinct membranous and cytoplasmic EpCAM expression in 51-80% of tumour cells. Scale bars correspond to 100 µm in panel (A) and 50 µm in panel (B). EpCAM: epithelial cell adhesion molecule; WDSCC: well-differentiated squamous cell carcinoma

**Figure 12:**
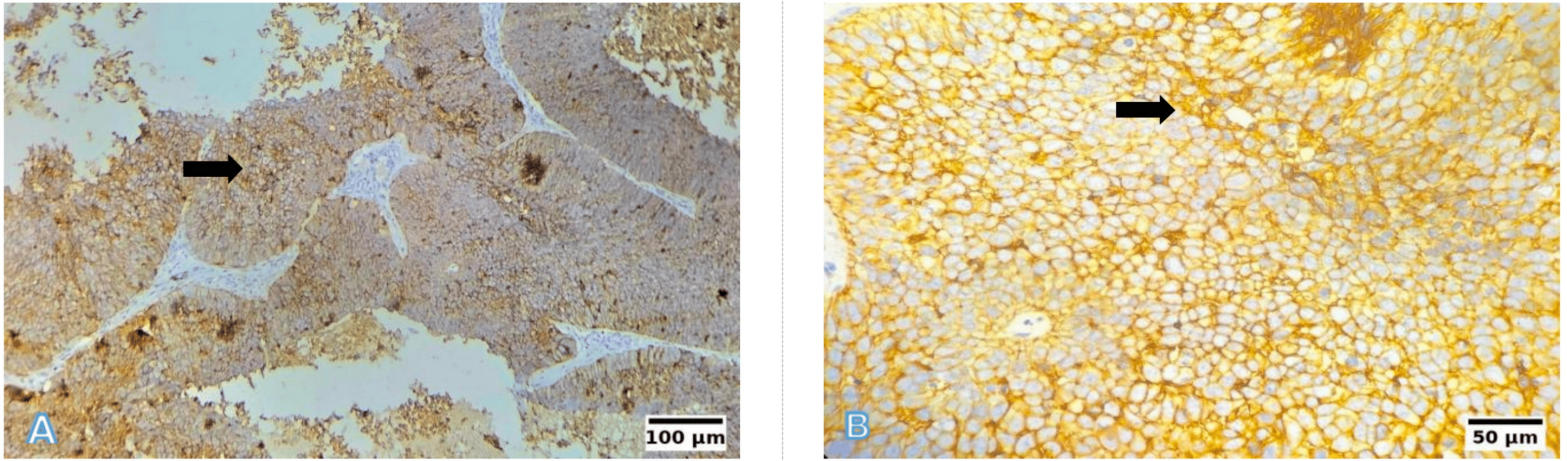
EpCAM immunohistochemistry: Overexpression (total score of 8; intensity 2 × proportion 4) (A) 10×: Strong EpCAM expression in >80% of tumour cells in MDSCC. (B) 40×: Intense membranous and cytoplasmic EpCAM positivity. Scale bars correspond to 100 µm in panel (A) and 50 µm in panel (B). EpCAM: epithelial cell adhesion molecule; MDSCC: moderately differentiated SCC

**Figure 13:**
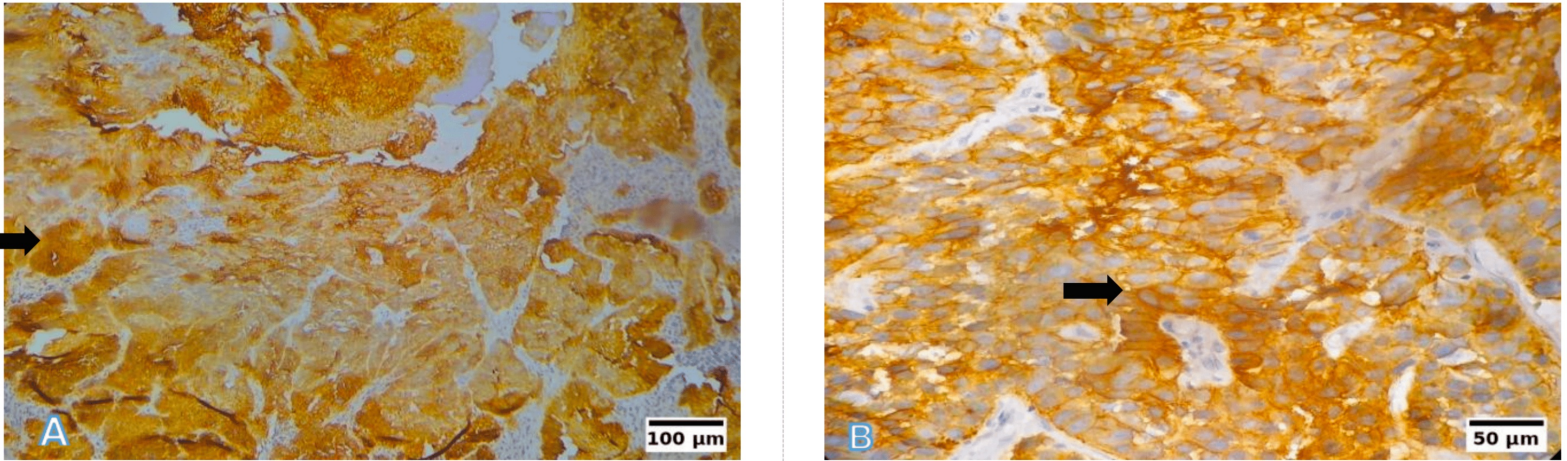
EpCAM immunohistochemistry: Overexpression (total score of 12; intensity 3 × proportion 4) (A) 10×: Strong diffuse EpCAM overexpression in PDSCC involving >80% of tumour cells. (B) 40×: Intense circumferential membranous and cytoplasmic staining. Scale bars correspond to 100 µm in panel (A) and 50 µm in panel (B). EpCAM: epithelial cell adhesion molecule; PDSCC: poorly differentiated SCC

EpCAM immunohistochemical expression patterns are demonstrated in Figures 9-13. The absence of EpCAM expression is shown in Figure 9, while weak expression with focal membranous staining is illustrated in Figure 10. Moderate-to-strong EpCAM overexpression is depicted in Figures 11-13, with increasing staining intensity and proportion of positive tumour cells. 

## Discussion

This study evaluated EpCAM immunohistochemical expression in 35 biopsy-confirmed OSCC cases from Northeast India and assessed its associations with various clinicopathological parameters. The overall overexpression rate of 28.6% is consistent with previously reported ranges in the literature, including studies by Sen et al. and others [8-10]. The concordance across geographically distinct cohorts having different risk profiles suggests that EpCAM overexpression in approximately a quarter to one-third of OSCC may represent a biologically intrinsic feature of a subset of oral carcinomas, rather than being driven solely by population-specific carcinogen exposures.

The demographic profile of our cohort - male predominance (60%), peak age of 51-60 years, and buccal mucosa as the most frequent site - reflects patterns characteristic of tobacco- and betel nut-associated OSCC in South Asia [12]. The male-to-female ratio of 1.5:1, while consistent with published Indian series, is lower than Western data, likely reflecting the higher prevalence of smokeless tobacco and areca nut use among women in Northeast India [1,5].

In our cohort, EpCAM overexpression appeared more frequent among patients reporting alcohol use compared to those reporting smokeless tobacco use. However, exposure variables were recorded in a binary manner without information on duration, intensity, or cumulative exposure, limiting meaningful comparison between exposure groups. The observed pattern may be hypothesis-generating but should be interpreted with caution. While alcohol has been proposed to enhance mucosal permeability and facilitate carcinogen penetration [13,14], and areca nut-related carcinogenesis involves oxidative stress and EGFR/MAPK pathway activation [15], the present data are insufficient to establish exposure-specific effects on EpCAM expression.

The significantly higher EpCAM overexpression in poorly differentiated (100%) compared to moderately differentiated (55.6%) and well-differentiated (12.5%) OSCC (p = 0.008) is comparable to the data of Sen et al. (PDSCC: 100%, MDSCC: 65%, WDSCC: 8.1%) and is consistent with the known biology of EpCAM in carcinogenesis [9]. EpCAM overexpression promotes epithelial-mesenchymal transition (EMT) by providing adhesion-independent signalling through EpICD nuclear activity and may sustain CSC populations in poorly differentiated tumours [7]. This association between histological grade and EpCAM expression was further supported by effect size analysis, which demonstrated that moderately and poorly differentiated tumours had higher odds of EpCAM overexpression compared to well-differentiated tumours (OR = 12.25; 95% CI: 2.18-68.6), although the wide confidence interval reflects the limited sample size. The 100% overexpression observed in poorly differentiated tumours, albeit based on only two cases, provides a basis for hypothesis generation and warrants validation in larger prospective studies.

Tongue carcinomas showed the highest numbers of EpCAM overexpression (57.1%), despite buccal mucosa being the most common site overall. This is concordant with Yanamoto et al., who reported EpCAM overexpression in 62.5% of tongue carcinomas and demonstrated its significant association with tumour size, lymph node metastasis, and histological differentiation [16]. The ventrolateral tongue is relatively thin and non-keratinised, which may contribute to increased susceptibility to invasion [1].

EpCAM represents an established therapeutic target, with adecatumumab and several others already under active clinical investigation [7]. The identification of a subgroup of OSCC-predominantly higher-grade, alcohol-associated, and tongue-sited tumours with consistent EpCAM overexpression may help define candidates for such targeted interventions.

Several limitations of this study must be acknowledged. First, the relatively small sample size limits the statistical power and generalisability of the findings, and certain subgroups - particularly poorly differentiated tumours - were underrepresented. Second, TNM clinical staging data were not available, as all specimens were obtained from incisional or punch biopsies rather than surgical resections, precluding assessment of the relationship between EpCAM expression and disease stage, and limiting evaluation of its potential prognostic significance. Third, as a single-centre study, the cohort may not fully represent the heterogeneity of the OSCC population across Northeast India, where risk factor profiles may vary across ethnic communities and geographic subregions. Fourth, survival and follow-up data were not available, precluding evaluation of the prognostic significance of EpCAM expression in terms of disease-free or overall survival. Fifth, carcinogenic exposures (smokeless tobacco use, smoking, and alcohol consumption) were recorded as broad categorical variables without detailed quantification of duration, frequency, or cumulative exposure, limiting the ability to assess dose-response relationships and constraining etiological interpretation. Finally, molecular correlates of EpCAM overexpression, including gene expression analysis, were beyond the scope of this study but would meaningfully augment the biological interpretation of the findings in future investigations.

## Conclusions

This pilot study demonstrates that EpCAM overexpression occurs in approximately 28.6% of OSCC cases in a Northeast Indian cohort, predominantly in poorly differentiated tumours, tongue carcinomas, and those cases that reported alcohol use. The statistically significant association of EpCAM overexpression with histological grade (p = 0.008) is further supported by effect size analysis, with moderately and poorly differentiated tumours demonstrating substantially higher odds of overexpression (OR = 12.25; 95% CI: 2.18-68.6); however, these findings should be interpreted with caution given the small sample size and require validation in larger cohort.

Preliminary trends linking EpCAM overexpression to male sex and reported alcohol exposure warrant evaluation in adequately powered studies. A multi-centre prospective study with a minimum cohort of 150-200 cases, incorporating TNM staging, survival follow-up, and molecular correlates (*EpCAM *gene expression, cancer stem cell markers), is recommended to validate the prognostic significance of EpCAM in OSCC and to evaluate its utility as a candidate target for anti-EpCAM therapeutic strategies in this population.
